# High-capacity device-independent quantum secure direct communication based on hyper-encoding

**DOI:** 10.1016/j.fmre.2023.11.006

**Published:** 2023-11-30

**Authors:** Hui Zeng, Ming-Ming Du, Wei Zhong, Lan Zhou, Yu-Bo Sheng

**Affiliations:** a College of Science, Nanjing University of Posts and Telecommunications, Nanjing 210023, China; b College of Electronic and Optical Engineering, & College of Flexible Electronics (Future Technology), Nanjing University of Posts and Telecommunications, Nanjing 210023, China; c Institute of Quantum Information and Technology, Nanjing University of Posts and Telecommunications, Nanjing 210003, China

**Keywords:** Quantum cryptography, Device-independent, Quantum secure direct communication, Hyper-encoding, Hyperentangled Bell state measurement, Secret message capacity

## Abstract

Quantum secure direct communication (QSDC) can directly transmit secret messages through quantum channel without keys. Device-independent (DI) QSDC guarantees the message security relying only on the observation of the Bell-inequality violation, but not on any detailed description or trust of the devices’ inner workings. Compared with conventional QSDC, DI-QSDC has relatively low secret message capacity. To increase DI-QSDC’s secret messages capacity, we propose a high-capacity DI-QSDC protocol based on the hyper-encoding technique. The total message leakage rate of our DI-QSDC protocol only relies on the most robust degree of freedom. We provide the numerical simulation of its secret message capacity altered with the communication distance. Our work serves as an important step toward the further development of DI-QSDC systems.

## Graphical abstract

**Figure:**
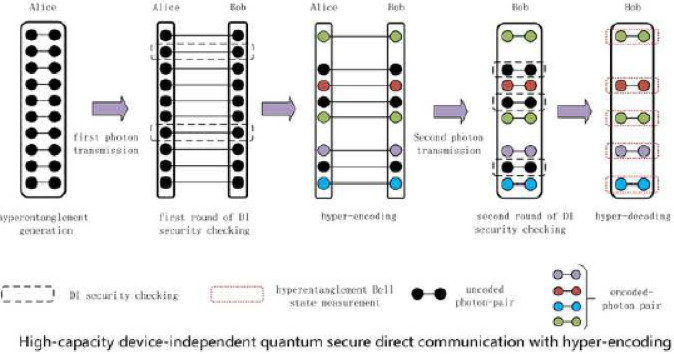

## 1 Introduction

Quantum secure communication is based on the basic principles of quantum mechanics such as the Heisenberg uncertainty and the no-cloning theorem. The theoretical unconditional security is its biggest advantage compared with classical communication, which can even resist the rapid progress of quantum computing [1], [2], [3], [4]. Quantum secure communication originates from the quantum key distribution (QKD) in 1984, which is used to distribute secure keys between two distant parties [5], [6]. So far, QKD has achieved great development in both theory and experiment [7], [8], [9], [10], [11], [12], [13], [14], [15]. Besides QKD, there are some important branches in the quantum secure communication, such as quantum secret sharing (QSS) [16], [17], [18], [19], [20] and quantum secure direct communication (QSDC) [21], [22], [23], [24], [25], [26], [27], [28], [29], [30], [31], [32], [33], [34], [35], [36], [37], [38], [39], [40], [41]. QSS enables a dealer to split a key into several parts and send each part to a participant. The participants can obtain the distributed keys only by cooperation. Different from QKD and QSS, QSDC can directly transmit secure messages through quantum channels without keys.

The first QSDC protocol was proposed by the group of Long [21]. In 2003 and 2004, the typical entanglement-based two-step QSDC protocol and single-photon-based QSDC protocol were successively proposed [22], [23]. These two typical QSDC protocols were demonstrated experimentally in 2016 and 2017, respectively [25], [26]. In 2017, the first long-distance QSDC experiment was realized [27]. During the past few years, QSDC has achieved great progress in both experiment and theory. In experiment aspect, a 15-user QSDC network with any two users being 40 km apart was reported in 2021 [34]. In 2022, the QSDC over 100 km fiber was realized [35]. Later, QSDC was adopted in the quantum networks with secure classical repeaters, which can offer the secure end-to-end communication across the entire network [40]. In theory, the complete security analysis of the practical QSDC was implemented in 2019 [28]. Later, the device-independent (DI) and measurement-device-independent (MDI) QSDC protocols were proposed, which can enhance QSDC’s security under practical experimental condition [29], [30]. In 2022, Sheng et al. proposed the one-step QSDC protocol, which reduces the photon transmission rounds from two to one, and thus can effectively simplify the operations and reduce the message loss [36]. Later, the DI and MDI one-step QSDC protocols were also proposed to increase one-step QSDC’s security with practical experimental devices [37], [38].

Similar to DI-QKD [42], [43], [44], [45], [46], [47], [48], [49], [50], [51], [52], DI-QSDC treats all experimental devices as black boxes. It ensures the communication security based solely on the observed data conclusively violating the Bell inequality (the Clauser-Horne-Shimony-Holt (CHSH) inequality), but not on any detailed description or trust of the inner workings of the experimental devices [42], [43]. In this way, DI-QSDC can resist all possible attacks on practical experimental devices. The original DI-QSDC protocol adopts the polarization degree of freedom (DOF) to encode messages [29]. Compared with conventional QSDC protocols, the original DI-QSDC protocol has relatively low secret message capacity. Hyper-encoding, which means encoding independently in multiple DOFs of a single photon, is a promising method to increase the capacity of single photon. Hyper-encoding serves as a good testing platform for quantum information experiments, such as implementing quantum logic gates [53], [54], adding control to arbitrary quantum operations [55], and implementing the high-capacity quantum secure communication [56], [57], [58] and quantum repeaters [59], [60].

In this paper, we propose a high-capacity DI-QSDC protocol with the hyper-encoding technique. The message sender prepares the polarization-spatial-mode hyperentangled photon pairs and encodes messages independently in both DOFs. The hyperentangled photon pairs are sent to the receiver by two rounds of photon transmission. The security of both photon transmission processes is guaranteed by the DI security checking in both DOFs [61], [62], [63]. Finally, the message receiver reads out the encoded messages in both DOFs by performing the hyperentanglement Bell-state measurement (HBSM). Our protocol has important application in the future quantum secure communication field.

The structure of this paper is as follows. In Section 2, we explain the high-capacity DI-QSDC protocol in detail. In Section 3, we perform the security analysis and simulate the secret message capacity of the high-capacity DI-QSDC protocol. In Section 4, we make some discussion and draw a conclusion.

## 2 The high-capacity DI-QSDC protocol with hyper-encoding

**Fig. 1:**
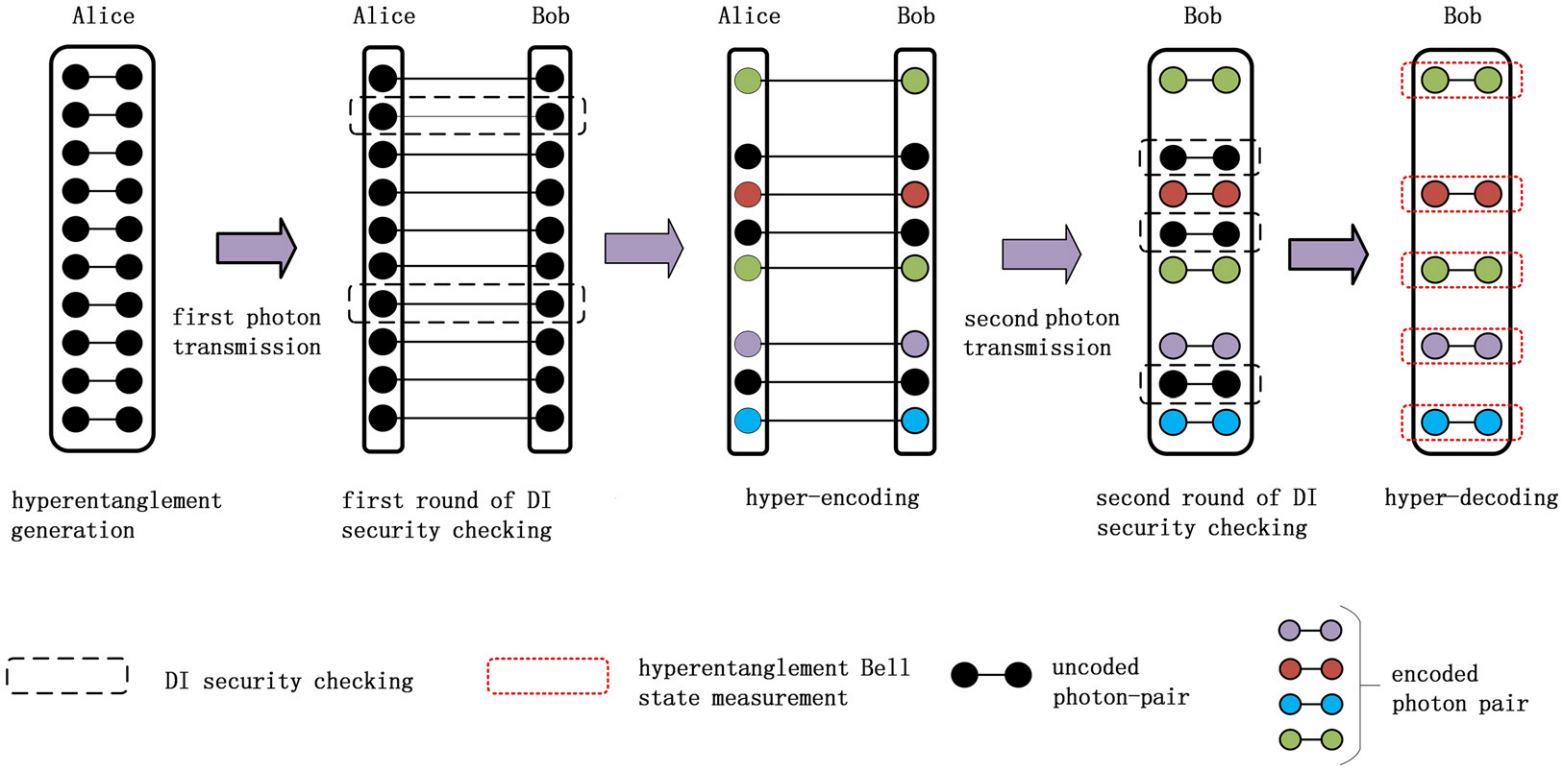
**The schematic diagram of the high-capacity DI-QSDC protocol with the hyper-encoding technique.** (a) hyperentanglement generation, (b) first round of DI security checking, (c) hyper-encoding, (d) second round of DI security checking, (e) hyper-decoding.

The basic principle of the high-capacity DI-QSDC protocol is shown in Fig. 1. Our protocol can be described as follows.

**Step 1** Alice prepares N identical polarization-spatial-mode hyperentangled photon pairs in $|\Phi^{+}\rangle =|\phi_{p}^{+}\rangle ⊗|\phi_{s}^{+}\rangle$ (N is large). Here, $|\phi_{p}^{+}\rangle$ and $|\phi_{s}^{+}\rangle$ belong to four Bell states in the polarization and spatial-mode DOFs, respectively, which can be written as:

(1) $$\begin{array}{cc} |\phi_{p}^{\pm }\rangle =\frac{1}{\sqrt{2}}(|\mathrm{HH}\rangle \pm \left|\mathrm{VV}\rangle \right) & \;\;\;\;|\phi_{s}^{\pm }\rangle =\frac{1}{\sqrt{2}}(|a_{1}a_{1}^{{}^{\text{'}}}\rangle \pm |a_{2}a_{2}^{{}^{\text{'}}}\rangle )\\ |\psi_{p}^{\pm }\rangle =\frac{1}{\sqrt{2}}(|\mathrm{HV}\rangle \pm \left|\mathrm{VH}\rangle \right) & \;\;\;\;|\psi_{s}^{\pm }\rangle =\frac{1}{\sqrt{2}}(|a_{1}a_{2}^{{}^{\text{'}}}\rangle \pm |a_{2}a_{1}^{{}^{\text{'}}}\rangle ) \end{array}$$

Here, the subscripts “p” and “s” represent the polarization and spatial-mode DOFs, respectively. In the polarization DOF, |H〉 (|V〉) represents the horizontal (vertical) polarization. Alice divides the N hyperentangled photon pairs into two photon sequences, including the checking (C) photon sequence $\left[C_{1},C_{2},C_{3},\ldots ,C_{N}\right]$ and the message (M) photon sequence $\left[M_{1},M_{2},M_{3},\ldots ,M_{N}\right]$. $a_{1}$ and $a_{2}$ are different spatial modes in the M photon sequence, while $a_{1}^{{}^{\prime }}$ and $a_{2}^{{}^{\prime }}$ represent different spatial modes in the C photon sequence.

**Step 2** Alice sends all the photons in the C sequence to Bob through the quantum channel and stores all the M photons into the quantum memory devices. $a_{1}^{{}^{\prime }}$ and $a_{2}^{{}^{\prime }}$ modes correspond to $b_{1}$ and $b_{2}$ modes in Bob’s location, respectively. After the photon transmission, the new four Bell states in the spatial-mode DOF shared by Alice and Bob are described as:

(2) $$\begin{array}{cc} |\phi_{\mathrm{sAB}}^{+}\rangle =\frac{1}{\sqrt{2}}(|a_{1}b_{1}\rangle +|a_{2}b_{2}\rangle ) & \;\;\;\;\;\;\;\;\;|\phi_{\mathrm{sAB}}^{-}\rangle =\frac{1}{\sqrt{2}}(|a_{1}b_{1}\rangle -|a_{2}b_{2}\rangle )\\ |\psi_{\mathrm{sAB}}^{+}\rangle =\frac{1}{\sqrt{2}}(|a_{1}b_{2}\rangle +|a_{2}b_{1}\rangle ) & \;\;\;\;\;\;\;\;\;|\psi_{\mathrm{sAB}}^{-}\rangle =\frac{1}{\sqrt{2}}(|a_{1}b_{2}\rangle -|a_{2}b_{1}\rangle ) \end{array}$$

After Bob receives the photons, he stores the received photons in the quantum memory devices.

**Step 3** Bob randomly selects a subset of photons from the received C sequence as the security-checking photons and announces their positions through a classical channel. Then, both parties extract the security-checking photons from quantum memory devices and perform the first round of DI security checking in both DOFs. In detail, in the polarization (spatial-mode) DOF, Alice randomly chooses one of the four measurement-basis {$A_{0p(s)}=\sigma_{zp(s)}$, $A_{1p(s)}=\left(\sigma_{zp(s)}+\sigma_{xp(s)}\right)/2$, $A_{2p(s)}=\left(\sigma_{zp(s)}-\sigma_{xp(s)}\right)/2$, $A_{3p(s)}=\sigma_{xp(s)}$ } to measure each of the security-checking photons, while Bob randomly chooses two measurement-basis {$B_{1p(s)}=A_{0p(s)}$, $B_{2p(s)}=A_{3p(s)}$} to measure each of his photons. Here, $\sigma_{z}$ and $\sigma_{x}$ represent the Pauli operators. The measurement results corresponding to the measurement bases are represented by:

(3) $$\begin{array}{c} a_{p}=\left\{a_{0p},a_{1p},a_{2p},a_{3p}\right\}\;b_{p}=\left\{b_{1p},b_{2p}\right\}\\ a_{s}=\left\{a_{0s},a_{1s},a_{2s},a_{3s}\right\}\;b_{s}=\left\{b_{1s},b_{2s}\right\} \end{array}$$

where $a_{ip(s)}$, $b_{jp(s)}\in \left\{+1,-1\right\}$, (i=0,1,2,3 and j=1,2). If a party obtains an inconclusive result (neither of the photon detectors response), the measurement result is randomly set as “+1” or “–1”. After all the security-checking photon pairs are measured, Alice and Bob publish their measurement bases and measurement results in both DOFs.

**Fig. 2:**
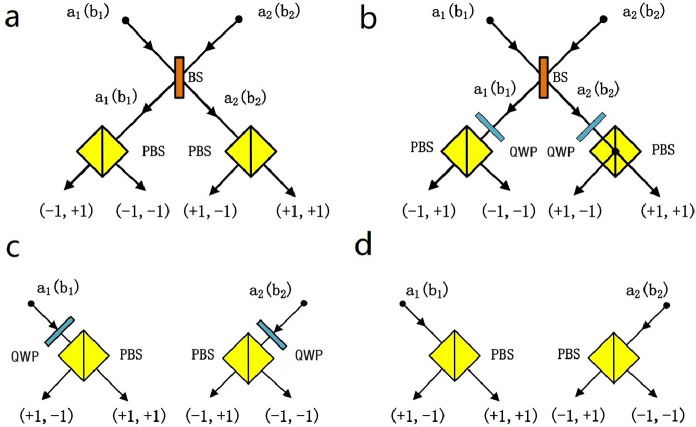
**Four linear optical apparatuses for Alice (Bob) measuring their photons and the corresponding results “+1” and “–1”, respectively**[62]. (a) $\sigma_{xs}\sigma_{zp}$ measurement bases, (b) $\sigma_{xs}\sigma_{xp}$ measurement bases, (c) $\sigma_{zs}\sigma_{xp}$ measurement bases, (d) $\sigma_{zs}\sigma_{zp}$ measurement bases. The polarization beam splitter (PBS) can totally transmit the photon in |H〉 and reflect the photon in |V〉. The 50:50 beam splitter (BS) is used to perform the Hadamard (H) operation in the spatial-mode DOF as $|i_{1}\rangle \to \frac{1}{\sqrt{2}}(|i_{1}\rangle +|i_{2}\rangle )$ and $|i_{2}\rangle \to \frac{1}{\sqrt{2}}(|i_{1}\rangle -|i_{2}\rangle )$(i=a,b). The quarter wave plate (QWP) is used to perform the H operation in the polarization DOF as $|H\rangle \to \frac{1}{\sqrt{2}}(|H\rangle +\left|V\rangle \right)$ and $|V\rangle \to \frac{1}{\sqrt{2}}(|H\rangle -\left|V\rangle \right)$.

The nonlocality test protocol for polarization-spatial-mode hyperentangled photon pairs with linear optical elements was proposed in 2003 [62] and experimentally demonstrated in 2005 [63]. In Fig. 2, we show four linear optical apparatuses for Alice (Bob) measuring the photon in $\sigma_{xs}\sigma_{zp}$, $\sigma_{xs}\sigma_{xp}$, $\sigma_{zs}\sigma_{xp}$, $\sigma_{zs}\sigma_{zp}$ bases and the corresponding measurement results “+1” and “–1”, respectively [62].

In the first case, in each DOF, when Alice selects $A_{1}$ or $A_{2}$, and Bob selects $B_{1}$ or $B_{2}$, their measurement results are used to estimate the CHSH polynomials as [42], [43]:

(4) $$\begin{array}{cc} & S_{1p}=\langle a_{1p}b_{1p}\rangle +\langle a_{1p}b_{2p}\rangle +\langle a_{2p}b_{1p}\rangle -\langle a_{2p}b_{2p}\rangle \\ & S_{1s}=\langle a_{1s}b_{1s}\rangle +\langle a_{1s}b_{2s}\rangle +\langle a_{2s}b_{1s}\rangle -\langle a_{2s}b_{2s}\rangle \end{array}$$

Here, $\langle a_{ip(s)}b_{jp(s)}\rangle$ are defined as the probability (P) of $a_{ip(s)}=b_{jp(s)}$ subtracting to that of $a_{ip(s)}\neq b_{jp(s)}$ (i=1,2 and j=1,2).

In the second case, in each DOF, when Alice selects $A_{0}$ and Bob selects $B_{1}$, the measurement results of both parties are used to estimate the bit-flip error rate ($Q_{B1}$) as [42], [43]:

(5) $$\begin{array}{c} Q_{B1p}=P\left(a_{0p}\neq b_{1p}\right)\;Q_{B1s}=P\left(a_{0s}\neq b_{1s}\right) \end{array}$$

In the third case, Alice selects $A_{3}$ and Bob selects $B_{2}$ in a DOF. The measurement results are used to estimate the phase-flip error rate ($Q_{P1}$) as:

(6) $$\begin{array}{c} Q_{P1p}=P\left(a_{3p}\neq b_{2p}\right)\;Q_{P1s}=P\left(a_{3s}\neq b_{2s}\right) \end{array}$$

In this way, we can obtain the total error rate ($Q_{1s}$ and $Q_{1p}$) in two DOFs after the first photon transmission as:

(7) $$\begin{array}{c} Q_{1p}=Q_{B1p}+Q_{P1p}\;Q_{1s}=Q_{B1s}+Q_{P1s} \end{array}$$

In the last case, when Alice selects $A_{0}$ and Bob selects $B_{2}$, or Alice selects $A_{3}$ and Bob selects $B_{1}$ in any DOF, both parties will discard the measurement results.

After the security checking, when the CHSH polynomials in both DOFs satisfy $2<S_{1p}\leq 2\sqrt{2}$ and $2<S_{1s}\leq 2\sqrt{2}$, the parties’ measurement results in both DOFs are non-locally correlated, and they can bound Eve’s photon interception rates in the polarization and spatial-mode DOFs with $S_{1p}$ and $S_{1s}$, respectively. In particularly, the maximal value $S_{1p}=2\sqrt{2}$ ($S_{1s}=2\sqrt{2}$) corresponds to the case that Alice and Bob share the maximally entangled state $|\phi_{p}^{+}\rangle$ ($|\phi_{sAB}^{+}\rangle$). In this case, Eve cannot eavesdrop any photon without being detected. As a result, when $2<S_{1p}\leq 2\sqrt{2}$ and $2<S_{1s}\leq 2\sqrt{2}$, the parties ensure the security of the first photon transmission process and go to the next step. On the other hand, if $S_{1p}\leq 2$ or $S_{1s}\leq 2$, the measurement results in the polarization or spatial-mode DOF are classically correlated. Under this case, there exists a trivial attack for Eve to eavesdrop photons without being detected, so that the photon transmission process is not secure and the parties have to discard the communication.

**Step 4** Alice extracts the photons in the M sequence from the quantum memory devices. Then, she randomly selects a subset of photons in the M photon sequence as the second round of security-checking photons, and does not perform any operation on them. For the rest photons, she encodes her messages on them by performing one of the four unitary operations in both DOFs. The four unitary operations in the polarization and spatial-mode DOFs can be written as:

(8) $$\begin{array}{cc} & U_{0p}=I=\left|H\rangle \langle H\right|+|V\rangle \langle V|\;U_{0s}=I=|a_{1}\rangle \langle a_{1}\left|+\right|a_{2}\rangle \langle a_{2}|\\ & U_{1p}=\sigma_{zp}=\left|H\rangle \langle H\right|-|V\rangle \langle V|\;U_{1s}=\sigma_{zs}=|a_{1}\rangle \langle a_{1}\left|-\right|a_{2}\rangle \langle a_{2}|\\ & U_{2p}=\sigma_{xp}=\left|H\rangle \langle V\right|+|V\rangle \langle H|\;U_{2s}=\sigma_{xs}=|a_{1}\rangle \langle a_{2}\left|+\right|a_{2}\rangle \langle a_{1}|\\ & U_{3p}=i\sigma_{yp}=\left|H\rangle \langle V\right|-|V\rangle \langle H|\;U_{3s}=i\sigma_{ys}=|a_{1}\rangle \langle a_{2}\left|-\right|a_{2}\rangle \langle a_{1}| \end{array}$$

**Fig. 3:**
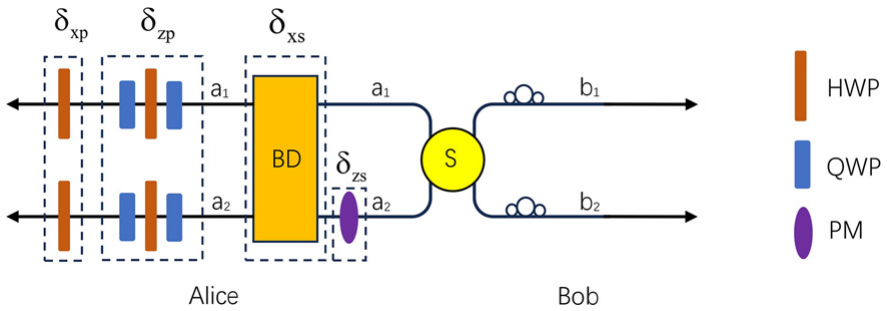
**The schematic diagram of the polarization-spatial-mode hyper-encoding in this high-capacity DI-QSDC protocol.** The beam displacer (BD) can swap the spatial-mode $a_{1}$ and $a_{2}$. The phase modulator (PM) can introduce a phase of π on the photon in $a_{2}$ mode. The half wave plate (HWP) can make |H〉→|V〉 and |V〉→|H〉, while the quarter wave plate (QWP) can make $|H\rangle \to \frac{1}{\sqrt{2}}(|H\rangle +\left|V\rangle \right)$ and $|V\rangle \to \frac{1}{\sqrt{2}}(|H\rangle -\left|V\rangle \right)$.

Here, $U_{0p}$, $U_{1p}$, $U_{2p}$, and $U_{3p}$ make $|\phi_{p}^{+}\rangle$ become $|\phi_{p}^{+}\rangle$, $|\phi_{p}^{-}\rangle$, $|\psi_{p}^{+}\rangle$, and $|\psi_{p}^{-}\rangle$, respectively, and $U_{0s}$, $U_{1s}$, $U_{2s}$, and $U_{3s}$ make $|\phi_{sAB}^{+}\rangle$ become $|\phi_{sAB}^{+}\rangle$, $|\phi_{sAB}^{-}\rangle$, $|\psi_{sAB}^{+}\rangle$, and $|\psi_{sAB}^{-}\rangle$, respectively. The schematic diagram of the polarization-spatial-mode hyper-encoding is shown in Fig. 3. In detail, in the spatial-mode DOF, Alice can perform the $\sigma_{xs}$ operation with the help of the beam displacer (BD), which can swap the spatial-mode $a_{1}$ and $a_{2}$. She can perform the $\sigma_{zs}$ operation by locating the phase modulator (PM) in the $a_{2}$ mode, which introduces a phase of π on the photon. By combining $\sigma_{xs}$ and $\sigma_{zs}$ operations, Alice can perform the $i\sigma_{ys}$ operation. In the polarization DOF, the $\sigma_{xp}$ and $\sigma_{zp}$ can be performed with the combination of the half wave plate (HWP) and the quarter wave plate (QWP). Similarly, Alice can perform the $i\sigma_{yp}$ operation by combining $\sigma_{xp}$ and $\sigma_{zp}$.

We define that $U_{0p(s)}$, $U_{1p(s)}$, $U_{2p(s)}$, $U_{3p(s)}$ represent the classical messages 00, 01, 10, and 11, respectively. In this way, one hyperentangled photon pair can encode 4 bits of messages. For avoiding Eve’s precisely interception during the second photon transmission process according to her intercepted photons in the first photon transmission process, after the encodings, Alice messes up the photons in the M photon sequence, and records the position of each photon in the original M sequence. Then, Alice sends the new M sequence to Bob. The $a_{1}$ and $a_{2}$ modes correspond to the $b_{1}^{{}^{\prime }}$ and $b_{2}^{{}^{\prime }}$ modes in Bob’s location, respectively.

**Step 5** After Bob receives the transmitted photons, he stores all photons in the quantum memory devices. Then, Alice announces the position of each photon in the original M sequence and the positions of the security-checking photons through a classical channel. Bob recovers the original M sequence and extracts the security-checking photons from the memory devices. Then, he performs the second round of DI security checking in two DOFs alone, which is the same as that in step 3 but only performed by Bob. After all the measurements, Bob can estimate the CHSH polynomials ($S_{2p}$ and $S_{2s}$) and the total error rate ($Q_{2p}$ and $Q_{2s}$). If the CHSH polynomials $S_{2p}$, $S_{2s}$ satisfy $2<S_{2p}\leq 2\sqrt{2}$ and $2<S_{2s}\leq 2\sqrt{2}$, the parties can ensure the security of the second photon transmission round and go to the next step. In contrary, if $S_{2p}\leq 2$ or $S_{2s}\leq 2$, the second photon transmission process is not secure and the parties have to discard the communication.

**Step 6** Bob extracts all the encoded hyperentangled photon pairs, and performs the HBSM on each photon pair. By comparing with the original hyperentangled state $|\Phi^{+}\rangle$, Bob can finally read out the encoded messages in both DOFs. If the HBSM obtains an inconclusive result caused by the photon loss, Bob will obtain a random result in each DOF.

## 3 Security analysis and the numerical simulation of the secret message capacity

Similar as DI-QKD, the security of the DI-QSDC protocol is based on a minimal set of fundamental assumptions. First, quantum physics is correct. Second, no unwanted information from Alice’s and Bob’s physical locations can be leaked outside. Meanwhile, we only require Eve to obey the laws of quantum physics. Eve can even control Alice’s hyperentanglement source and fabricate Alice’s and Bob’s measurement devices. In both security checking processes, Alice and Bob can only use the observed correlation between the measurement basis selection and the measurement results to bound Eve’s knowledge in each DOF. Here, we consider a general attack, the collective attack, where Eve applies the same attack on each transmitted photon in each DOF. After the photon transmission, all the photon pairs have the same form in each DOF. We also assume that in each DOF, the party’s measurement result is only a function of the measurement basis selection.

The secret message capacity $C_{S}$ in each DOF is defined as the amount of transmitted correct and secure qubits divided by the amount of the encoded photon pairs. It is noticed that although we have specified a particular state in each DOF to produce these correlations, we do not assume anything about the implementation of the correlations when computing $C_{S}$ in each DOF.

By considering the collective attacks, the secret message capacity from Alice to Bob in the polarization and spatial-mode DOFs are lower bounded by the Devetak-Winter rate as [42], [43]:

(9) $$\begin{array}{c} C_{Sp}\geq I{\left(A:B\right)}_{p}-I{\left(A:E\right)}_{p}\;C_{Ss}\geq I{\left(A:B\right)}_{s}-I{\left(A:E\right)}_{s} \end{array}$$

where $I{\left(A:B\right)}_{p(s)}$ and $I{\left(A:E\right)}_{p(s)}$ represent the mutual information between Alice and Bob, Alice and Eve in the polarization (spatial-mode) DOF, respectively.

In ideal devices and quantum channel condition, as an entangled photon pair in each DOF carries 2 bits of messages, it is obvious that $I{\left(A:B\right)}_{p}=I{\left(A:B\right)}_{s}=2$. After the ideal photon transmission processes, the encoded photon pairs are perfectly transmitted to Bob, which leads to $S_{1p(s)}=S_{2p(s)}=2\sqrt{2}$. In this way, we can obtain $I{\left(A:E\right)}_{p}=I{\left(A:E\right)}_{s}=0$. As a result, both $C_{Sp}$ and $C_{Ss}$ can reach the maximal value of 2 and the total secret message capacity $C_{St}=C_{Sp}+C_{Ss}=4$.

In the practical condition, the imperfect devices and quantum channel would reduce $S_{1p(s)}$ and $S_{2p(s)}$, and thus reduce $C_{Sp}$ ($C_{Ss}$). In detail, $I{\left(A:B\right)}_{p}$ and $I{\left(A:B\right)}_{s}$ depend on the total error rate $Q_{tp}$ and $Q_{ts}$, respectively. We can obtain [42], [43]

(10) $$\begin{array}{c} I{\left(A:B\right)}_{p}=2\left[1-h\left(Q_{tp}\right)\right]\;I{\left(A:B\right)}_{s}=2\left[1-h\left(Q_{ts}\right)\right] \end{array}$$

where h is the binary Shannon entropy with the form of:

(11) $$\begin{array}{c} h\left(x\right)=-x\log_{2}x-\left(1-x\right)\log_{2}\left(1-x\right) \end{array}$$

Here, we mainly consider the influence from noisy quantum channel. In this way, we consider the ideal quantum memory and photon detectors. In the DI-QSDC protocol, the photons transmit in the quantum channel twice. The environmental noise may cause the photon transmission loss and decoherence. In general, the transmission efficiency $\eta_{t}$ has the form of $\eta_{t}=10^{-\alpha d/10}$, where α=0.2 dB/km for the standard fiber and d is the communication distance. Meanwhile, we adopt the white noise model in each DOF. In this way, after the first photon transmission, Alice and Bob will share N pairs of mixed states $\rho_{1}=\rho_{p1}⊗\rho_{s1}$ as:

(12) $$\begin{array}{ccc} \rho_{p1} & = & \eta_{t}F_{p}\left|\phi_{p}^{+}\rangle \langle \phi_{p}^{+}\right|+\eta_{t}\frac{\left(1-F_{p}\right)}{3}\left(|\phi_{p}^{-}\rangle \langle \phi_{p}^{-}\left|+\right|\psi_{p}^{+}\rangle \langle \psi_{p}^{+}\left|+\right|\psi_{p}^{-}\rangle \langle \psi_{p}^{-}|\right)\\ & & +\;\frac{1}{2}\left(1-\eta_{t}\right){(|H\rangle }_{A}\langle H|+{|V\rangle }_{A}\langle V|) \end{array}$$

(13) $$\begin{array}{ccc} \rho_{s1} & = & \eta_{t}F_{s}\left|\phi_{\mathrm{sAB}}^{+}\rangle \langle \phi_{\mathrm{sAB}}^{+}\right|+\eta_{t}\frac{\left(1-F_{s}\right)}{3}(\left|\phi_{\mathrm{sAB}}^{-}\rangle \langle \phi_{\mathrm{sAB}}^{-}\right|+\left|\psi_{\mathrm{sAB}}^{+}\rangle \langle \psi_{\mathrm{sAB}}^{+}\right|\\ & & +\;\left|\psi_{\mathrm{sAB}}^{-}\rangle \langle \psi_{\mathrm{sAB}}^{-}\right|)+\frac{1}{2}\left(1-\eta_{t}\right)(|a_{1}\rangle \langle a_{1}\left|+\right|a_{2}\rangle \langle a_{2}|) \end{array}$$

where $F_{p}$ and $F_{s}$ are the fidelities of the target state $|\phi_{p}^{+}\rangle$ and $|\phi_{sAB}^{+}\rangle$, respectively. In this way, we can obtain the theoretical values of $S_{1p}$, $S_{1s}$, $Q_{1p}$, and $Q_{1s}$ as:

(14) $$\begin{array}{ccc} S_{1p} & = & 2\sqrt{2}\eta_{t}F_{p}\;S_{1s}=2\sqrt{2}\eta_{t}F_{s}\\ Q_{1p} & = & Q_{B1p}+Q_{P1p}=\frac{1}{2}\left(1-\eta_{t}\right)+\eta_{t}\left(1-F_{p}\right)\\ Q_{1s} & = & Q_{B1s}+Q_{P1s}=\frac{1}{2}\left(1-\eta_{t}\right)+\eta_{t}\left(1-F_{s}\right) \end{array}$$

After the second photon transmission process, the quantum states of the security checking photons are $\rho_{2}=\rho_{p2}⊗\rho_{s2}$ with the form of

(15) $$\begin{array}{ccc} \rho_{p2} & = & \eta_{t}^{2}F_{p}^{2}|\phi_{p}^{+}\rangle \langle \phi_{p}^{+}|+\frac{\eta_{t}^{2}\left(1-F_{p}^{2}\right)}{3}(|\phi_{p}^{-}\rangle \langle \phi_{p}^{-}\left|+\right|\psi_{p}^{+}\rangle \langle \psi_{p}^{+}\left|+\right|\psi_{p}^{-}\rangle \langle \psi_{p}^{-}|)\\ & & +\;\frac{\eta_{t}\left(1-\eta_{t}\right)}{2}{(|H\rangle }_{B}\langle H|+{|V\rangle }_{B}\langle V|)+\frac{\eta_{t}\left(1-\eta_{t}\right)}{2}{(|H\rangle }_{A}\langle H|+{|V\rangle }_{A}\langle V|)\\ & & +\;{\left(1-\eta_{t}\right)}^{2}\left|\mathrm{vac}\rangle \langle \mathrm{vac}\right|\\ \rho_{s2} & = & \eta_{t}^{2}F_{s}^{2}|\phi_{\mathrm{sAB}}^{+}\rangle \langle \phi_{\mathrm{sAB}}^{+}|+\frac{\eta_{t}^{2}\left(1-F_{s}^{2}\right)}{3}\left(\right|\phi_{\mathrm{sAB}}^{-}\rangle \langle \phi_{\mathrm{sAB}}^{-}\left|+\right|\psi_{\mathrm{sAB}}^{+}\rangle \langle \psi_{\mathrm{sAB}}^{+}|\\ & & +|\psi_{\mathrm{sAB}}^{-}\rangle \langle \psi_{\mathrm{sAB}}^{-}|)+\frac{\eta_{t}\left(1-\eta_{t}\right)}{2}(|b_{1}\rangle \langle b_{1}\left|+\right|b_{2}\rangle \langle b_{2}|)\\ & & +\frac{\eta_{t}\left(1-\eta_{t}\right)}{2}(|b_{1}\text{'}\rangle \langle b_{1}\text{'}|+|b_{2}\text{'}\rangle \langle b_{2}\text{'}|)+{\left(1-\eta_{t}\right)}^{2}\left|\mathrm{vac}\rangle \langle \mathrm{vac}\right| \end{array}$$

where |vac〉 means the vacuum state. In this way, the theoretical values of $S_{2p}$, $S_{2s}$, $Q_{2p}$, and $Q_{2s}$ can be written as:

(16) $$\begin{array}{ccc} S_{2p} & = & 2\sqrt{2}F_{p}^{2}\eta_{t}^{2}\;S_{2s}=2\sqrt{2}F_{s}^{2}\eta_{t}^{2}\\ Q_{2p} & = & Q_{B2p}+Q_{P2p}=\frac{1}{2}\left(1-\eta_{t}^{2}\right)+\eta_{t}^{2}\left(1-F_{p}^{2}\right)\\ Q_{2s} & = & Q_{B2s}+Q_{P2s}=\frac{1}{2}\left(1-\eta_{t}^{2}\right)+\eta_{t}^{2}\left(1-F_{s}^{2}\right) \end{array}$$

It is obvious that the total error rate in the polarization and spatial-mode DOFs satisfy $Q_{tp}=Q_{2p}$ and $Q_{ts}=Q_{2s}$.

Then, we calculate $I{\left(A:E\right)}_{p}$ and $I{\left(A:E\right)}_{s}$. After the first round of photon transmission, when $S_{1p}>2$ and $S_{1s}>2$, we can estimate the Holevo quantities in both DOFs by:

(17) $$\begin{array}{c} \chi_{S_{1p}}\leq h\left(\frac{1+\sqrt{{\left(S_{1p}/2\right)}^{2}-1}}{2}\right)\\ \chi_{S_{1s}}\leq h\left(\frac{1+\sqrt{{\left(S_{1s}/2\right)}^{2}-1}}{2}\right) \end{array}$$

It has been proved that in any DOF, Eve’s photon interception rate ($I_{E}$) equals to the Holevo quantity [42], [43]. In this way, in our hyper-encoding DI-QSDC protocol, we can obtain Eve’s photon interception rate during the first photon-transmission round equals to the minimum value of $\chi_{S_{1p}}$ and $\chi_{S_{1s}}$. Under practical experimental condition, since the spatial-mode entanglement has a stronger ability to resist noise than the polarization entanglement [64], [65], we can obtain $F_{s}>F_{p}$, so that $S_{1s}>S_{1p}$. From Eq. 17, it is obvious that $h\left(\frac{1+\sqrt{{\left(S_{1s}/2\right)}^{2}-1}}{2}\right)<h\left(\frac{1+\sqrt{{\left(S_{1p}/2\right)}^{2}-1}}{2}\right)$. In this way, Eve’s photon interception rate can be bounded by $h(\frac{1+\sqrt{{\left(S_{1s}/2\right)}^{2}-1}}{2})$.

As only when Eve intercepts both photons of a hyperentangled photon pair, can he finally read out the encoded messages through the HBSM. As a result, the message leakage rates $I{\left(A:E\right)}_{p}$ and $I{\left(A:E\right)}_{s}$ in the polarization and spatial-mode DOFs are bounded by Eve’s photon interception rate in the first photon transmission process, which can be written as:

(18) $$I{\left(A:E\right)}_{p}=I{\left(A:E\right)}_{s}=2\chi_{S_{1s}}\leq 2h\left(\frac{1+\sqrt{{\left(S_{1s}/2\right)}^{2}-1}}{2}\right)$$

$I{\left(A:E\right)}_{p(s)}$ can reach the maximal value of $2h(\frac{1+\sqrt{{\left(S_{1s}/2\right)}^{2}-1}}{2})$ only when Eve can intercept all the corresponding M photons of his intercepted C photons. From step 4 of Section 2, Alice messes up the photons in the M photon sequence before the second photon transmission process. When the photon number N is large, the probability that $I{\left(A:E\right)}_{p(s)}=2h\left(\frac{1+\sqrt{{\left(S_{1s}/2\right)}^{2}-1}}{2}\right)$ will be close to 0.

Based on above calculations, we can provide the lower bounded of the secret message capacity in the polarization and spatial-mode DOFs as:

(19) $$\begin{array}{c} C_{\mathrm{Sp}}\geq 2\left[1-h\left(Q_{2p}\right)-h\left(\frac{1+\sqrt{{\left(S_{1s}/2\right)}^{2}-1}}{2}\right)\right]\\ C_{\mathrm{Ss}}\geq 2\left[1-h\left(Q_{2s}\right)-h\left(\frac{1+\sqrt{{\left(S_{1s}/2\right)}^{2}-1}}{2}\right)\right] \end{array}$$

The total secret message capacity $C_{St}$ of our hyper-encoding DI-QSDC protocol can be written as:

(20) $$\begin{array}{ccc} C_{\mathrm{St}} & \geq & 2\left[1-h\left(Q_{2p}\right)-h\left(\frac{1+\sqrt{{\left(S_{1s}/2\right)}^{2}-1}}{2}\right)\right]\\ & & +\;2\left[1-h\left(Q_{2s}\right)-h\left(\frac{1+\sqrt{{\left(S_{1s}/2\right)}^{2}-1}}{2}\right)\right] \end{array}$$

Compared with the original DI-QSDC protocol with the secret message capacity of $C_{Sp}$, the adoption of the hyper-encoding technique can effectively increase DI-QSDC’s secret message capacity. In particular, as in practical experimental condition, $I{\left(A:E\right)}_{p}+I{\left(A:E\right)}_{s}=4\chi_{S_{1s}}<2\left[h\left(\frac{1+\sqrt{{\left(S_{1p}/2\right)}^{2}-1}}{2}\right)+h\left(\frac{1+\sqrt{{\left(S_{1s}/2\right)}^{2}-1}}{2}\right)\right]$, we can obtain $C_{St}>2C_{Sp}$.

**Fig. 4:**
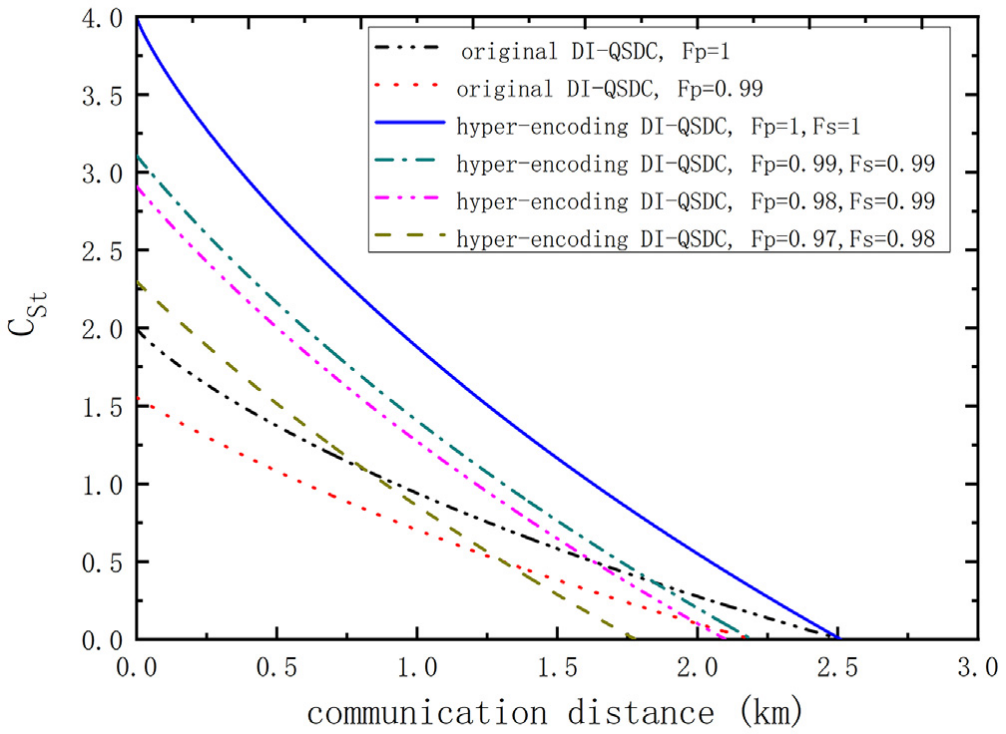
**The total secret message capacity**$C_{St}$**of our high-capacity DI-QSDC protocol altered with the communication distance *d*.** Here, we consider the ideal photon memory devices and photon detectors. We set four fidelity conditions, $F_{p}=F_{s}=1$, $F_{p}=F_{s}=0.99$, $F_{p}=0.98$ and $F_{s}=0.99$, $F_{p}=0.97$ and $F_{s}=0.98$, respectively.

We provide the numerical simulation of the total secrete message capacity $C_{St}$ altered with the communication distance din Fig. 4. Here, we consider the ideal photon memory devices and photon detectors and set four fidelity conditions, say, $F_{p}=F_{s}=1$, $F_{p}=F_{s}=0.99$, $F_{p}=0.98$ and $F_{s}=0.99$, $F_{p}=0.97$ and $F_{s}=0.98$. We also provide $C_{S}$ of the original DI-QSDC protocol [29] with $F_{p}=1$ and $F_{p}=0.99$, respectively. It can be found that $C_{St}$ reduces with the growth of communication distance and the reduction of the fidelities $F_{p}$ and $F_{s}$. The adoption of the hyper-encoding technique can efficiently increase the DI-QSDC’s secrete message capacity. We define the maximal communication distance dm corresponding to $C_{St}=0$. dm of our DI-QSDC with above four groups of fidelities are about 2.511 km, 2.196 km, 1.975 km, and 1.650 km, respectively. Here, we consider $F_{p}=F_{s}$ for simplicity. The threshold of $F_{P}$ ($F_{S}$) is about 0.925. If $F_{p}$ ($F_{s}$) in each DOF decreases below 0.925, no correct message can be transmitted from Alice to Bob. In addition, the adoption of the hyper-encoding DI-QSDC does not extend the maximal communication distance. For example, the values of dm corresponding to $F_{p}=F_{s}=1$ and $F_{p}=F_{s}=0.99$ equal to those of the original DI-QSDC protocol with $F_{p}=1$ and $F_{p}=0.99$, respectively.

## 4 Discussion

In this paper, we introduce the hyper-encoding technique into the DI-QSDC, which can increase the message capacity of each photon pair, and thus increase DI-QSDC’s secret message capacity. The entanglement in the polarization and spatial-mode DOFs are totally independent, so that we can perform the non locality verification and message encoding in each DOF independently. After the photon transmission, the error (decoherence) in one DOF only influences the non locality verification and encoding in this DOF, but does not influence that in the other DOF. As a result, the independent DOF encoding is flexible and has high efficiency in the non locality verification and message encoding. Meanwhile, besides polarization and spatial-mode DOFs, the photon has some other DOFs, such as the time-bin, the orbital angular momentum (OAM), and the frequency, which have been widely used in quantum secure communication field. Our protocol can be extended to adopt other two-DOF hyper-encoding or multi-DOF hyper-encoding, such as the polarization-time-energy hyper-encoding, polarization-spatial-mode-frequency hyper-encoding, the polarization-spatial-mode-time-bin hyper-encoding, and the polarization-time-bin-OAM hyper-encoding. In theory, the more DOFs one uses, the higher secret message capacity will achieve. It is worth noting that the total message leakage rate only depends on the CHSH polynomial in the most robust DOF. In this way, by adopting a robust DOF, we can effectively reduce the message leakage and increase the total secret message capacity.

From Eqs. 14–16, it is obvious that the photon transmission loss and decoherence would largely reduce the CHSH polynomials, and thus reduce $C_{St}$. We can adopt the heralded hyperentanglement amplification [66] after both photon transmission processes to solve the photon transmission loss problem. In detail, in the first photon transmission process, once Alice emits one photon, Bob performs the heralded amplification protocol. If the amplification protocol is successful, he will announce Alice to reserve the corresponding M photon. Otherwise, if the amplification protocol fails, he will announce Alice to discard the corresponding M photon. In the second photon transmission process, once Alice emits a single photon, Bob performs the heralded amplification protocol. If the amplification protocol is successful, he will announce Alice to encode her next photon. If the amplification protocol fails, Bob will discard the corresponding photon in the C sequence and tell Alice to perform the same encoding on the next photon, and so on, until Bob successfully receives the encoded photon. In this way, with the help of the heralded hyperentanglement amplification, the message loss problem can be completely solved. Meanwhile, we can adopt the hyperentanglement purification [67] after the first photon transmission process to resist the decoherence. Combined with the heralded hyperentanglement amplification and the hyperentanglement purification after the first photon transmission process, the parties can construct the nearly perfect hyperentanglement channel ($S_{1p}\to 2\sqrt{2}$ and $S_{1s}\to 2\sqrt{2}$). In this way, the security of the first photon transmission process can be guaranteed. As the message leakage in any DOF relies on the photon interception rate in the first photon transmission process, we can nearly eliminate the message leakage in both DOFs. However, after the second photon transmission process, the parties cannot use the hyperentanglement purification, for it may change the encoded messages in both DOFs. As a result, the decoherence occurring in the second photon transmission process still exists, which may cause the QBERs in each DOF with the form of

(21) $$\begin{array}{c} Q_{tp}=1-F_{p}\;Q_{ts}=1-F_{s} \end{array}$$

Based on above discussion, the adoption of the heralded hyperentanglement amplification and the hyperentanglement purification in the high-capacity DI-QSDC protocol can nearly eliminate the message leakage, effectively reduce the QBERs in both DOFs, and thus largely increase the maximal communication distance.

Finally, we discuss the experimental realization of our hyper-ending DI-QSDC protocol. Our protocol requires the hyperentangled photon pairs as the resources and hyperentanglement storage. The generation protocols of hyperentanglement in polarization, spatial-mode, time energy, time-bin, and OAM DOFs have been widely researched in experiments [68], [69], [70], [71], [72]. In addition, the distribution of the polarization-energy-time hyperentanglement via an intra-city free-space link was experimentally realized [73]. During the past few years, the hyperentanglement storage technology has developed rapidly. The polarization-energy-time and path-OAM hyperentangled photon pairs in solid-state quantum memory have been experimentally demonstrated, respectively [74], [75]. In particular, complete HBSM is the key element of the hyper-encoding DI-QSDC protocol, which can distinguish all the 16 possible hyperentangled Bell states. After ensuring the security of photon transmission, Bob requires to perform the HBSM on each hyper-encoded photon pair to read out the encoded messages in both DOFs. The existing complete HBSM protocols all reply on the nonlinear optical elements, such as the cross-Kerr nonlinearity [76]. Although cross-Kerr nonlinearity is still challenging to realize under current experimental condition, it has made great progresses in experiment during past few years [77], [78], [79], [80]. Actually, our high-capacity DI-QSDC protocol can also use the partial HBSM, such as that in Ref. [81]. As shown in Table 1, that partial HBSM protocol can divide the 16 possible polarization-spatial-mode hyperentangled Bell states into 14 distinguishable groups with the help of the time-bin DOF. The first 12 groups of hyperentangled Bell states can be distinguished. In the 13th and 14th groups, Charlie can only determine the Bell states in the spatial-mode DOF, but cannot distinguish $|\phi_{p}^{\pm }\rangle$. In this way, the adoption of the partial HBSM may influence the message recoding of our protocol. For guaranteeing the correctness of the transmitted messages, if the partial HBSM result may cause error in the polarization DOF with the probability of $\frac{1}{4}$, the BSM result and the encoded bits in the polarization DOF would be discarded. In this way, some encoded messages in the polarization DOF may be lost due to the partial HBSM. For guaranteeing the message integrity, Alice has to record the positions of the lost messages and encode the lost messages on the photon pairs in the next communication round.

**Table 1:** **The distinction of the polarization-spatial-mode hyperentangled Bell states**[81].

Group	State	Group	State
1	$|\phi_{p}^{+}\rangle ⊗|\phi_{s}^{+}\rangle$	8	$|\psi_{p}^{-}\rangle ⊗|\psi_{s}^{-}\rangle$
2	$|\phi_{p}^{-}\rangle ⊗|\phi_{s}^{+}\rangle$	9	$|\psi_{p}^{+}\rangle ⊗|\phi_{s}^{-}\rangle$
3	$|\phi_{p}^{-}\rangle ⊗|\phi_{s}^{-}\rangle$	10	$|\psi_{p}^{+}\rangle ⊗|\phi_{s}^{+}\rangle$
4	$|\phi_{p}^{+}\rangle ⊗|\phi_{s}^{-}\rangle$	11	$|\psi_{p}^{-}\rangle ⊗|\phi_{s}^{+}\rangle$
5	$|\psi_{p}^{+}\rangle ⊗|\psi_{s}^{-}\rangle$	12	$|\psi_{p}^{-}\rangle ⊗|\phi_{s}^{-}\rangle$
6	$|\psi_{p}^{+}\rangle ⊗|\psi_{s}^{+}\rangle$	13	$|\phi_{p}^{\pm }\rangle ⊗|\psi_{s}^{-}\rangle$
7	$|\psi_{p}^{-}\rangle ⊗|\psi_{s}^{+}\rangle$	14	$|\phi_{p}^{\pm }\rangle ⊗|\psi_{s}^{+}\rangle$

## 5 Conclusion

In conclusion, DI-QSDC can resist all possible attacks from the imperfect experimental devices and thus guarantee the security of the transmitted messages under practical imperfect experimental condition. To increase DI-QSDC’s secret message capacity, in this paper, we propose a polarization-spatial-mode hyper-encoding DI-QSDC protocol. In our protocol, the photons should be transmitted in the quantum channel twice. The security of each photon transmission process is guaranteed by the observation of data conclusively violating the CHSH inequality in each DOF. Our protocol is unconditionally secure in theory. The hyper-encoding can effectively increase the message capacity of each photon pair and thus increase the total secret message capacity. Our hyper-encoding DI-QSDC protocol can be extended to use the hyper-encoding in other two DOFs or the multi-DOF hyper-encoding. The total message leakage rate only depends on the CHSH polynomial in the most robust DOF. We numerically simulate the total secret message capacity as a function of the communication distance. The maximal communication distance of our DI-QSDC with $F_{p}=F_{s}=1$ is about 2.511 km and the threshold values of $F_{p}$ and $F_{s}$ are about 0.925. Moreover, with the help of the heralded hyperentanglement amplification and hyperentanglement purification, we can largely increase the maximal communication distance and nearly eliminate the message leakage in both DOFs. Based on above features, this hyper-encoding DI-QSDC protocol may have potential applications in the future quantum communication field.

## Declaration of competing interest

The authors declare that they have no conflicts of interest in this work.

## References

[1] Lau J.W.Z., Lim K.H., Shrotriya H. (2022). NISQ computing: Where are we and where do we go?. AAPPS Bull..

[2] Chen Z., Sun Z., Pei Y.K. (2022). Generalized sparse codes for non-gaussian channels: Code design, algorithms, and applications. Funda. Res..

[3] Lu B., Liu L., Song J.Y. (2023). Recent progress on coherent computation based on quantum squeezing. AAPPS Bull..

[4] Huang J.S., Chen X.J., Li X.D. (2023). Chip-based photonic graph states. AAPPS Bull..

[5] Bennett C.H., Brassard G. (1984). Proc. IEEE Int. Conf. on Com. Sys. and Signal Proc..

[6] Ekert A.K. (1991). Quantum cryptography based on Bell’s theorem. Phys. Rev. Lett..

[7] Xu F.H., Ma X.F., Zhang Q. (2020). Secure quantum key distribution with realistic devices. Rev. Mod. Phys..

[8] Chen Y.A., Zhang Q., Chen T.Y. (2021). An integrated space-to-ground quantum communication network over 4600 km. Nature.

[9] Liu W.B., Li C.L., Xie Y.M. (2021). Homodyne detection quadrature phase shift keying continuous-variable quantum key distribution with high excess noise tolerance. PRX Quant..

[10] Liu B., Xia S., Xiao D. (2022). Decoy-state method for quantum-key-distribution-based quantum private query. Sci. China Phys. Mech. Astron..

[11] Gu J., Cao X.Y., Fu Y. (2022). Experimental measurement-device-independent type quantum key distribution with flawed and correlated sources. Sci. Bull..

[12] Liang K.X., Chai G., Cao Z.W. (2023). Bayesian parameter estimation for continuous-variable quantum key distribution. Phys. Rev. Appl..

[13] Xie Y.M., Bai J.L., Lu Y.S. (2023). Advantages of asynchronous measurement-device-independent quantum key distribution in intercity networks. Phys. Rev. Appl..

[14] Liu Y., Zhang W.J., Jiang C. (2023). Experimental twin-field quantum key distribution over 1000 km fiber distance. Phys. Rev. Lett..

[15] Xie Y.M., Weng C.X., Lu Y.S. (2023). Scalable high-rate twin-field quantum key distribution networks without constraint of probability and intensity. Phys. Rev. A.

[16] Hillery M., Buek V., Berthiaume A. (1999). Quantum secret sharing. Phys. Rev. A.

[17] Bell B., Markham D., Herrera-Mart D. (2014). Experimental demonstration of graph-state quantum secret sharing. Nat. Commun..

[18] Fu Y., Yin H.L., Chen T.Y. (2015). Long-distance measurement-device-independent multiparty quantum communication. Phys. Rev. Lett..

[19] Ju X.X., Zhong W., Sheng Y.B. (2022). Measurement-device-independent quantum secret sharing with hyper-encoding. Chin. Phys. B.

[20] Shen A., Cao X.Y., Wang Y. (2023). Experimental quantum secret sharing based on phase encoding of coherent states. Sci. China Phys. Mech. Astron..

[21] Long G.L., Liu X.S. (2002). Theoretically efficient high-capacity quantum-key-distribution scheme. Phys. Rev. A.

[22] Deng F.G., Long G.L., Liu X.S. (2003). Two-step quantum direct communication protocol using the Einstein-Podolsky-Rosen pair block. Phys. Rev. A.

[23] Deng F.G., Long G.L. (2004). Secure direct communication with a quantum one-time pad. Phys. Rev. A.

[24] Wang C., Deng F.G., Li Y.S. (2005). Quantum secure direct communication with high-dimension quantum superdense coding. Phys. Rev. A.

[25] Hu J.Y., Yu B., Jing M.Y. (2016). Experimental quantum secure direct communication with single photons. Light Sci. Appl..

[26] Zhang W., Ding D.S., Sheng Y.B. (2017). Quantum secure direct communication with quantum memory. Phys. Rev. Lett..

[27] Zhu F., Zhang W., Sheng Y.B. (2017). Experimental long-distance quantum secure direct communication. Sci. Bull..

[28] Qi R.Y., Sun Z., Lin Z.S. (2019). Implementation and security analysis of practical quantum secure direct communication. Light Sci. Appl..

[29] Zhou L., Sheng Y.B., Long G.L. (2020). Device-independent quantum secure direct communication against collective attacks. Sci. Bull..

[30] Zhou Z.R., Sheng Y.B., Niu P.H. (2020). Measurement-device-independent quantum secure direct communication. Sci. China Phys. Mech. Astron..

[31] Long G.L., Zhang H.R. (2021). Drastic increase of channel capacity in quantum secure direct communication using masking. Sci. Bull..

[32] Liu X., Li Z.J., Luo D. (2021). Practical decoy-state quantum secure direct communication. Sci. China Phys. Mech. Astron..

[33] Cao Z.W., Wang L., Liang K.X. (2021). Continuous-variable quantum secure direct communication based on Gaussian mapping. Phys. Rev. Appl..

[34] Qi Z.T., Li Y.H., Huang Y.W. (2021). A 15-user quantum secure direct communication network. Light Sci. Appl..

[35] Zhang H.R., Sun Z., Qi R.Y. (2022). Realization of quantum secure direct communication over 100 km fiber with time-bin and phase quantum states. Light Sci. Appl..

[36] Sheng Y.B., Zhou L., Long G.L. (2022). One-step quantum secure direct communication. Sci. Bull..

[37] Zhou L., Sheng Y.B. (2022). One-step device-independent quantum secure direct communication. Sci. China Phys. Mech. Astron..

[38] Ying J.W., Zhou L., Zhong W. (2022). Measurement-device-independent one-step quantum secure direct communication. Chin. Phys. B.

[39] Zhou L., Xu B.W., Zhong W. (2023). Device-independent quantum secure direct communication with single-photon sources. Phys. Rev. Appl..

[40] Long G.L., Pan D., Sheng Y.B. (2022). An evolutionary pathway for the quantum internet relying on secure classical repeaters. IEEE Netw..

[41] Liang K.X., Cao Z.W., Chen X.L. (2023). A quantum secure direct communication scheme based on intermediate-basis. Front. Phys..

[42] Acín A., Brunner N., Gisin N. (2007). Device-independent security of quantum cryptography against collective attacks. Phys. Rev. Lett..

[43] Pironio S., Acín A., Brunner N. (2009). Device-independent quantum key distribution secure against collective attacks. New J. Phys..

[44] Lim C.C.W., Portmann C., Tomamichel M. (2013). Device-independent quantum key distribution with local bell test. Phys. Rev. X.

[45] Arnon-Friedman R., Dupuis F., Fawzi O. (2018). Practical device-independent quantum cryptography via entropy accumulation. Nat. Commun..

[46] Kołodyński J., Máttar A., Skrzypczyk P. (2020). Device-independent quantum key distribution with single-photon sources. Quantum.

[47] Das S., Bäuml S., Winczewski M. (2021). Universal limitations on quantum key distribution over a network. Phys. Rev. X.

[48] Kaur E., Horodecki K., Das S. (2022). Upper bounds on device-independent quantum key distribution rates in static and dynamic scenarios. Phys. Rev. Appl..

[49] Horodecki K., Winczewski M., Das S. (2022). Fundamental limitations on the device-independent quantum conference key agreement. Phys. Rev. A.

[50] Liu W.Z., Zhang Y.Z., Zhen Y.Z. (2022). Toward a photonic demonstration of device-independent quantum key distribution. Phys. Rev. Lett..

[51] Zhang W., van Leent T., Redeker K. (2022). A device-independent quantum key distribution system for distant users. Nature.

[52] Nadlinger D.P., Drmota P., Nichol B.C. (2022). Experimental quantum key distribution certified by Bell’s theorem. Nature.

[53] Lanyon B.P., Barbieri M., Almeida M.P. (2009). Simplifying quantum logic using higher-dimensional hilbert spaces. Nat. Phys..

[54] Micuda M., Sedlak M., Straka I. (2013). Efficient experimental estimation of fidelity of linear optical quantum Toffoli gate. Phys. Rev. Lett..

[55] Zhou X.Q., Ralph T.C., Kalasuwan P. (2011). Adding control to arbitrary unknown quantum operations. Nat. Commun..

[56] Graham T.M., Bernstein H.J., Wei T.C. (2015). Superdense teleportation using hyperentangled photons. Nat. Commun..

[57] Hu X.M., Guo Y., Liu B.H. (2023). Progress in quantum teleportation. Nat. Rev. Phys..

[58] Chapman J.C., Lim C.C.W., Kwiat P.G. (2022). Hyperentangled time-bin and polarization quantum key distribution. Phys. Rev. Appl..

[59] Starek R., Mikova M., Straka I. (2018). Experimental realization of SWAP operation on hyper-encoded qubits. Opt. Express.

[60] Huang C.X., Hu X.M., Guo Y. (2022). Entanglement swapping and quantum correlations via symmetric joint measurements. Phys. Rev. Lett..

[61] Chen Z.B., Zhang Q., Bao X.H. (2006). Deterministic and efficient quantum cryptography based on Bell’s theorem. Phys. Rev. A.

[62] Chen Z.B., Pan J.W., Zhang Y.D. (2003). All-versus-nothing violation of local realism for two entangled photons. Phys. Rev. Lett..

[63] Yang T., Zhang Q., Zhang J. (2005). All-versus-nothing violation of local realism by two-photon, four-dimensional entanglement. Phys. Rev. Lett..

[64] Simon C., Pan J.W. (2002). Polarization entanglement purification using spatial entanglement. Phys. Rev. Lett..

[65] Huang C.X., Hu X.M., Liu B.H. (2022). Experimental one-step deterministic polarization entanglement purification. Sci. Bull..

[66] Yang G., Zhang Y.S., Yang Z.R. (2019). Linear-optical heralded amplification protocol for two-photon spatial-mode-polarization hyperentangled state. Quant. Inform. Process..

[67] Ren B.C., Du F.F., Deng F.G. (2014). Two-step hyperentanglement purification with the quantum-state-joining method. Phys. Rev. A.

[68] Barreiro J.T., Langford N.K., Peters N.A. (2005). Generation of hyperentangled photon pairs. Phys. Rev. Lett..

[69] Prilmüller M., Huber T., Müller M. (2018). Hyperentanglement of photons emitted by a quantum dot. Phys. Rev. Lett..

[70] Shi X.F. (2021). Hyperentanglement of divalent neutral atoms by Rydberg blockade. Phys. Rev. A.

[71] Yasir P.A.A., Chandrashekar C.M. (2022). Generation of hyperentangled states and two-dimensional quantum walks using j or q plates and polarization beam splitters. Phys. Rev. A.

[72] Zhao P., Yang M.Y., Zhu S. (2023). Generation of hyperentangled state encoded in three degrees of freedom. Sci. China-Phys. Mech. Astron..

[73] Steinlechner F., Ecker S., Fink M. (2017). Distribution of high-dimensional entanglement via an intra-city free-space link. Nat. Commun..

[74] Tiranov A., Lavoie J., Ferrier A. (2015). Storage of hyperentanglement in a solid-state quantum memory. Optica.

[75] Zhang W., Ding D.S., Dong M.X. (2016). Experimental realization of entanglement in multiple degrees of freedom between two quantum memories. Nat. Commun..

[76] Sheng Y.B., Deng F.G., Long G.L. (2010). Complete hyperentangled-bell-state analysis for quantum communication. Phys. Rev. A.

[77] Hoi I.C., Kockum A.F., Palomaki T. (2013). Giant cross-kerr effect for propagating microwaves induced by an artificial atom. Phys. Rev. Lett..

[78] Beck K.M., Hosseini M., Duan Y. (2016). Large conditional single-photon cross-phase modulation. PNAS.

[79] Tiarks D., Schmidt S., Rempe G. (2016). Optical π phase shift created with a single-photon pulse. Sci. Adv..

[80] Sinclair J., Angulo D., Thompson K. (2022). Measuring the time atoms spend in the excited state due to a photon they do not absorb. PRX Quant..

[81] Gao C.Y., Ren B.C., Zhang Y.X. (2020). Universal linear-optical hyperentangled bell-state measurement. Appl. Phys. Express.

